# Cue-Induced Brain Activation in Chronic Ketamine-Dependent Subjects, Cigarette Smokers, and Healthy Controls: A Task Functional Magnetic Resonance Imaging Study

**DOI:** 10.3389/fpsyt.2018.00088

**Published:** 2018-03-21

**Authors:** Yanhui Liao, Maritza Johnson, Chang Qi, Qiuxia Wu, An Xie, Jianbin Liu, Mei Yang, Maifang Huang, Yan Zhang, Tieqiao Liu, Wei Hao, Jinsong Tang

**Affiliations:** ^1^Department of Psychiatry, The Second Xiangya Hospital, Central South University, Changsha, China; ^2^Mental Health Institute, The Second Xiangya Hospital, Central South University, Changsha, China; ^3^National Clinical Research Center on Mental Disorders, Changsha, China; ^4^National Technology Institute on Mental Disorders, Changsha, China; ^5^Hunan Key Laboratory of Psychiatry and Mental Health, Changsha, China; ^6^Department of Psychiatry and Biobehavioral Sciences, University of California, Los Angeles, Los Angeles, CA, United States; ^7^Department of Radiology, The People’s Hospital of Hunan Province, Changsha, China; ^8^Department of Addiction Medicine, Shenzhen Mental Health Center, Shenzhen Kangning Hospital, Shenzhen, China; ^9^Kangda Voluntary Drug Rehabilitation Center, Changsha, China

**Keywords:** ketamine users, cigarette smokers, cue, brain activation, functional magnetic resonance imaging

## Abstract

**Background:**

Observations of drug-related cues may induce craving in drug-dependent patients, prompting compulsive drug-seeking behavior. Sexual dysfunction is common in drug users. The aim of the study was to examine regional brain activation to drug (ketamine, cigarette smoking) associated cues and natural (sexual) rewards.

**Methods:**

A sample of 129 [40 ketamine use smokers (KUS), 45 non-ketamine use smokers (NKUS) and 44 non-ketamine use non-smoking healthy controls (HC)] participants underwent functional magnetic resonance imaging (fMRI) while viewing ketamine use related, smoking and sexual films.

**Results:**

We found that KUS showed significant increased activation in anterior cingulate cortex and precuneus in response to ketamine cues. Ketamine users (KUS) showed lower activation in cerebellum and middle temporal cortex compared with non-ketamine users (NKUS and HC) in response to sexual cues. Smokers (KUS and NKUS) showed higher activation in the right precentral frontal cortex in response to smoking cues. Non-ketamine users (NKUS and HC) showed significantly increased activation of cerebellum and middle temporal cortex while viewing sexual cues.

**Conclusion:**

These findings clearly show the engagement of distinct neural circuitry for drug-related stimuli in chronic ketamine users. While smokers (both KUS and NKUS) showed overlapping differences in activation for smoking cues, the former group showed a specific neural response to relevant (i.e., ketamine-related) cues. In particular, the heightened response in anterior cingulate cortex may have important implications for how attentionally salient such cues are in this group. Ketamine users (KUS) showed lower activation in response to sexual cues may partly reflect the neural basis of sexual dysfunction.

## Introduction

Over the last decade, ketamine has increasingly become a more widely used recreational drug among young people (1, 2). Chronic ketamine use is associated with cognitive changes (2), and our previous studies suggest that these changes are accompanied by marked brain changes that correlate with the magnitude of its use (3, 4).

Drug craving is thought to be a powerful motivational state or a very strong desire that drives the ketamine user to seek ketamine. For example, one subject recruited to our study reported that in the absence of available ketamine she would sniff white powder shaved from a wall. Undoubtedly, craving is clearly an important facet of chronic ketamine use. However, the mechanisms of drug craving in chronic ketamine users are not fully understood. Given that ketamine abuse is a growing problem, which is often accompanied by serious adverse effects such as ketamine-induced ulcerative cystitis, kidney dysfunction, psychosis, depression, cognitive impairment, and neurological changes (2), it becomes exceedingly important to explore and understand how craving drives ketamine or other drug users to use drugs without consideration of negative consequences. One way to address this question is the use of functional magnetic resonance imaging (fMRI) to characterize brain responses to drug-related cues that induce craving (5).

To date, there is a line of evidence indicating that nicotine, alcohol, cocaine, and other drugs of abuse are associated with the activation of some specific brain regions. This fMRI drug-related cue-induced brain activation is often associated with treatment outcomes and relapse (6). For example, in cigarette smokers, fMRI studies indicate smoking-related cue-induced brain activation predominantly in the prefrontal cortex, anterior cingulate cortex, ventral striatum, amygdala, hippocampus, and thalamus (7–13). In addition, smoking cessation selectively reduced responses to smoking cues in the amygdala (14); in heavy drinkers and individuals with alcohol use disorders, a meta-analysis paper demonstrated alcohol cues elicited robust activation in the limbic and prefrontal regions, and showed greater activation in the parietal and temporal regions when compared to controls. Furthermore, cue-elicited ventral striatum activation was most frequently correlated with behavioral measurements and activation in this region often reduced by treatment (15). However, in reviewing the literature, studies using fMRI to examine the effects of cue exposure on different drugs showed mixed findings, making it difficult to identify the reliable patterns of activation in a particular sample or specific drug-related cue exposure paradigm.

In order to characterize ketamine use and cigarette smoking-related cue-induced brain activation, we presented two types of addictive drug cues, ketamine and nicotine, during fMRI to investigate brain activation patterns to ketamine or smoking cues in chronic ketamine users (also smokers), cigarette smokers, and non-ketamine use non-smoking healthy controls (HC).

Based on previous cue-activation studies and behavior studies in illicit and non-illicit substance users, we hypothesized that ketamine use cue-elicited craving of brain activation will be stronger and wider than that of smoking cue-elicited. Also, based on results from our and other previous studies of brain structure changes in prefrontal cortex and anterior cingulate cortex in chronic ketamine users (3, 4, 16) and smoking cue-induced brain-imaging studies (17), we hypothesized that compared with non-ketamine use non-smoking HC, substance use subjects [ketamine use smokers (KUS) and non-ketamine use smokers (NKUS)] would have increased activation in prefrontal cortex and anterior paralimbic structures during ketamine-specific cue and smoking-specific cue presentations.

Loss of sexual interest or pleasure is a common symptom for drug abusers. For example, from a sample of 1,076 substance abusers, 45.2% had been suffering from sexual dysfunctions (18); out of 701 drug abusers, 36.4% reported erectile dysfunction (19); additionally, there is a higher prevalence of sexual dysfunction in female ketamine abusers with cystitis when compared with ketamine abusers without cystitis (20). Thus, we also presented non-addictive but salient cues in the form of sexual stimuli, which are known to induce activation in the hypothalamus, thalamus, amygdala, anterior cingulate gyrus (ACC), insula, fusiform gyrus, precentral gyrus, parietal cortex, and occipital cortex in healthy people (21), and reduce brain responses to sexual stimuli in the anterior cingulate and dorsolateral prefrontal cortex in breast cancer survivors with chemotherapy (22). Evidence shows that chronic illicit drug abusers (including ketamine abusers) commonly demonstrate sexual dysfunction and a previous study showed that cocaine users had smaller activation than the comparison subjects when shown a sex film (23). Given this, we hypothesized that levels of sexual cue-induced brain activation would be lower in chronic ketamine users (KUS) than that in non-ketamine users (NKUS and HC).

## Materials and Methods

### Subjects

One hundred and twenty-nine subjects 129 (40 chronic ketamine and nicotine-dependent subjects/KUS, 45 otherwise healthy nicotine-dependent subjects/NKUS, and 44 non-ketamine use non-smoking HC) were recruited in this study. All subjects were Han Chinese, aged 19–39 with normal or corrected-to-normal vision. Ketamine dependent volunteers were recruited from the Kangda Voluntary Drug Rehabilitation Centers in the Hunan Province and the Department of Addiction Medicine, Hunan Brain Hospital. All ketamine use subjects met the Diagnostic and Statistical Manual of Mental Disorders, fourth edition (DSM-IV) criteria for lifetime ketamine dependence determined from the Structured Clinical Interview (SCID) (24). Ketamine use subjects were excluded if they met criteria for other substance dependence (excluding nicotine dependence; all ketamine dependent subjects smoked at least eight cigarettes for more than 1 year and met DSM-IV criteria for nicotine dependence) at any time. The smokers and non-smokers were screened to ensure that they had no past neurological or psychiatric history. Smokers who had smoked 10 cigarettes per day or more during the previous year and had no period of smoking abstinence longer than 3 months in the past year, and met DSM-IV criteria for nicotine dependence were eligible for this study. All non-smokers in this sample reported no history of smoking behavior in the past. Subjects were excluded if they reported: major medical or psychiatric disorders, current use of psychotropic medications, use of intravenous drugs, pregnancy, and contraindications for MRI. None of the participants reported daily consumption of alcohol, and none reported experiencing social consequences secondary to alcohol use, or any history with difficulty ceasing alcohol intake. Subjects were required to abstain from ketamine for at least 48 h and nicotine for at least 12 h before scanning and from other psychoactive substances for at least 2 weeks. Nicotine patches were provided as needed. A licensed psychiatrist, at MD level, conducted all clinical interviews. The protocol was approved by the university ethics committee (The Second Xiangya Hospital of Central South University Review Board, No. S054, 2008) and the studies were carried out in accordance with the Declaration of Helsinki. Subjects were fully informed about the measurement and MRI scanning procedures in the study. Written informed consent was given and obtained by all subjects.

Ketamine craving and smoking craving were assessed by The Visual Analog Scale for Craving (VASc) (25). The VASc displays a scale from 0 to 10, where 0 represents null craving and 10 represents the most extreme craving.

### Stimuli and Design

Three 2-min sexual-related visual films selected from Asian movies of heterosexual activity, which contained hugs, kisses, and sexual acts; three 2-min ketamine-related films made by ketamine user, which visually showed the process of snorting ketamine; and three 2-min smoking-related films made by otherwise healthy smokers, which contained the process of cigarette smoking (Figure S1 in Supplementary Material).

All films intentionally were devoid of other appetitive stimuli (e.g., alcohol, food, tea, caffeine, gaming, etc.). Every film was presented using a Linux laptop computer with in-house stimulus delivery software for 2 min, followed at random by a black screen for 30 s. Additionally, stimulus order was randomized and no stimulus was repeated during the experiments. Together, all films lasted 22 min and 30 s using an Epson (Long Beach, CA, USA) MP 7200 LCD projector onto a screen placed at the feet of the MRI scanner bed and was viewed using a mirror mounted on the head coil.

### Imaging Acquisition and Preprocessing

Neuroimaging was conducted using a 3T Siemens Trio MRI scanner. The protocol began with initial structural scans followed by a series of functional runs during which participants completed the ketamine, cigarette, and sexual cues tasks. Structural T1-weighted images were acquired in a sagittal orientation employing a magnetization prepared rapid gradient-echo sequence with the following parameters: slice thickness = 1 mm, gap = 0 mm, repetition time = 2,000 ms, echo time = 2.6 ms, field of view = 256 cm × 256 cm, flip angle = 8°, matrix size = 256 × 256, and slices = 176. Functional MRI data were obtained using a gradient-echo echo-planar imaging (GRE-EPI) sequence with the following parameters: TR/TE = 2,000/30 ms, matrix = 64 × 64, flip angle = 90°, FOV = 220 mm × 220 mm, 32 interleaved axial slices, thickness = 3 mm, slice gap = 1 mm. The first three volumes of each scan were discarded to allow for T1 equilibrium effects.

Imaging analysis was done using SPM5 (Wellcome Trust Centre for Neuroimaging, London, United Kingdom). Images were corrected for the acquisition time delay between different slices and then realigned to the first volume for head-motion correction. Functional images were then normalized according to standard co-registration procedures using the individual’s structural scan. Then, all realigned and normalized images were smoothed with an 8 mm × 8 mm × 8 mm full width half maximum Gaussian filter. To remove low-frequency signal drift, a high-pass filter (with cutoff frequency 1/120 Hz) was applied.

### Statistical Analysis

Statistical Analysis of MRI data were conducted using SPM5 (Wellcome Trust Centre for Neuroimaging, London, UK). For each subject, fMRI responses were modeled using a canonical hemodynamic response function. The general linear model was used to perform a first level, within-participant analysis on the functional data from each subject individually for the primary contrasts: ketamine minus ketamine baseline, cigarette minus cigarette baseline, and sexual minus sexual baseline. Estimated motion correction parameters were included as additional covariates. Within-group effects were tested using single-sample *t* tests on contrast images for each group separately. Between-group differences were tested using an ANOVA test with variances assumed unequal between groups. Age and sex were included as covariates. Clusters were considered as significant if they reached a combined voxel-extend threshold of an uncorrected voxel level of *p* < 0.001 and cluster extent >10 voxels, as determined based on Monte Carlo simulation with AlphaSim correlation to *p* < 0.005. In order to test the relationship between drug craving scales (VASc) and cue-induced brain activation, ROI based analysis was done according to the fMRI findings.

Demographic and clinical variables analyses were conducted using Statistical Package for Social Sciences (SPSS) version 16. ANOVA test were used for comparison of demographic variables and cognitive tests. Two sample *t* tests were used for comparing the mean smoking variables and drug craving values between ketamine users and smokers.

## Results

### Participant Characteristics

All subjects were Han Chinese and were characterized typically by upper-middle-income socioeconomic status. The three groups were well-matched in age, gender, handedness, and marriage status. However, levels of education were not quite matched for the three groups. Smoking craving scales in smokers group is stronger than that of ketamine users. Detailed demographic and clinical characteristics for the three groups have been reported previously (26) and are also summarized in Table 1.

**Table 1:** Demographic and drug use characteristics of patients with ketamine dependence, chronic smokers and HC subjects.

	Ketamine users/smokers (*n* = 40)	Smokers (*n* = 45)	Non-smokers (*n* = 44)	ANOVA, *F* test	Two sample*t* test
**Demographic variables**					
Age, years, mean ± SD	26.8 (4.93)	27.9 (5.60)	26.3 (5.84)	*F* = 0.99 *p* = 0.373	
Range (years)	19–39	19–39	19–38		
Male/female	32/8	37/8 (17.78%)	34/10 (22.7%)		
Subjects’ education, years, mean ± SD	11.9 ± 2.8	13.1 ± 2.96	15.0 ± 2.6	*F* = 13.22 *p* = 0.000	
Right/left-handed	39/1	43/2	43/1		
Unmarried/married	25/15	26/19	29/15		

**Ketamine use variables**					
Age of first use, years, mean ± SD	23.10 ± 5.21	–	–		
Range (years)	14–36	–	–		
Duration, months, mean ± SD	41.7 ± 21.58	–	–		
Range (months)	12–126	–	–		
Times of using ketamine/day	1.85	–	–		
Range (times)	1–4	–	–		
Quantity of using ketamine/time (g)	0.77	–	–		
Range (g)	0.1–2.5	–	–		

**Smoking variables**					
Age of first smoking, years, mean ± SD	15.5 ± 3.70	18.0 ± 4.25	–		*p* = 0.004
Range (years)	10–30	11–30			
Duration, years, mean ± SD	11.4 ± 4.95	10.2 ± 5.76	–		*p* = 0.303
Range (years)	1.5–21	1.5–21	–		
Smoked cigarette/day, mean ± SD	16.5 ± 7.79	20.3 ± 7.61	–		*p* = 0.031
Range (cigarettes)	8–40	10–40	–		

**Drug craving**					
Ketamine craving (cm)	6.14 ± 2.81	–	–		
Smoking craving (cm)	5.36 ± 2.28	6.41 ± 1.72	–		*p* = 0.016

### Functional Brain Activation Analyses of Ketamine Use, Smoking, and Sexual-Related Short Films

**Figure 1:**
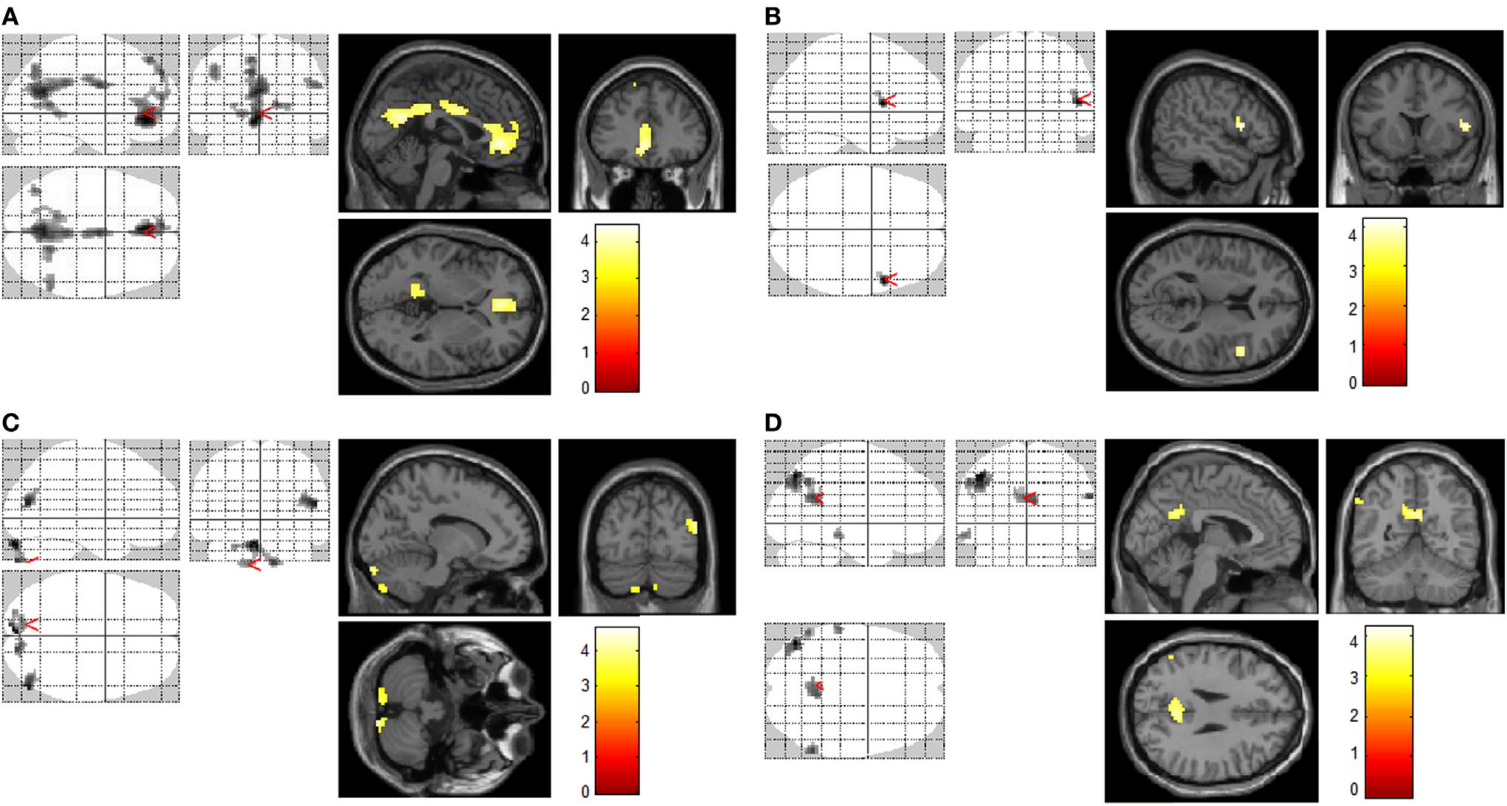
Increased brain regions of activation by different cues. Increased brain regions of activation in chronic ketamine users during exposure to ketamine use-related films when compared with control subjects [smokers and non-smokers **(A)**]; increased brain regions of activation in chronic smokers (chronic ketamine users were also chronic smokers) during exposure to smoking-related films when compared with non-smokers **(B)**; increased brain regions of activation in control subjects (smokers and non-smokers) during exposure to sexual films when compared with chronic ketamine users **(C)**; in respects to ketamime cue minus smoking cue, increased brain regions of activation in chronic ketamine users when compared with chronic smokers **(D)**. AlphaSim corrected *p* < 0.005.

Our primary interest was to compare brain activation, elicited in response to different types of addictive substances [i.e., ketamine and nicotine in ketamine users and non-ketamine users (smokers, non-smokers)] (see Table 2 and Figure 1). Another interest was to compare substance use and sexual cue-induced brain activation in substance users (ketamine users, smokers) and control subjects. For ketamine use related films and sexual films, there was no significant difference of brain activation between smokers and non-smokers; similarly, for smoking-related films no statistical significance of brain activation between ketamine users (also smokers) and smokers was shown (alphaSim corrected *p* < 0.005). We, therefore, performed between-group comparisons for activity during each condition. In order to remove the effects of gender, we also recalculated the results using male subjects only (see Figure S2 in Supplementary Material).

**Table 2:** Regions of brain activation during exposure to ketamine use related films (40 ketamine users VS 45 smokers + 44 non-smokers), smoking (40 ketamine users/also smokers + 45 smokers VS 44 non-smokers), and sexual cue (45smokers + 44 non-smokers VS 40 ketamine users).

Cue	Anatomical region	Cluster size (no. voxel)	Voxel level *p* uncorrected	Peak T value	Coordinates (mm)			Voxel*z* value
					*x*	*y*	*z*	
Ketamine use-related cue (K > S + N)	Left anterior cingulate cortex	378	0.000	4.45	−3	39	−6	4.28
	Precuneus	361	0.000	4.19	0	−60	24	4.05
	Cingulate gyrus	77	0.000	3.96	0	−6	30	3.84
	Left inferior parietal cortex	53	0.000	3.76	−42	−69	45	3.66
	Right posterior cingulate	45	0.000	3.76	18	−54	9	3.66
	Left occipital cortex (lingual gyrus)	56	0.000	3.63	−15	−48	0	3.53
	Right parietal cortex (supramarginal gyrus)	53	0.000	3.62	57	−54	27	3.52
Smoking-related cue (K + S > N)	Righ frontal cortex (precentral gyrus)	33	0.000	4.20	51	12	9	4.06
Sexual cue (K < S + N)	Left cerebellum	123	0.000	4.65	−6	−90	−24	4.46
	Middle temporal cortex	80	0.000	4.33	54	−75	18	4.17
Ketamine cue minus smoking cue (K > S)	Left inferior parietal cortex	130	0.000	4.22	−45	−69	48	4.14
	Posterior congulate/precuneus	81	0.000	3.74	6	−51	24	3.68
	Left middle temporal cortex	30	0.000	3.58	−63	−30	−12	3.53

*K, ketamine use group (ketamine users were also smokers, *n* = 40); S, smoking group (*n* = 45); N, non-smoking group (*n* = 44)*.

*AlphaSim corrected p < 0.005*.

#### Functional Activation Analyses: Ketamine Craving

In response to ketamine use-related films, we observed greater brain regions of activation in multiple and extensive regions including the left anterior cingulate cortex, precuneus, cingulate gyrus, left inferior parietal cortex, right posterior cingulate, left occipital cortex (lingual gyrus), and right parietal cortex (supramarginal gyrus) in KUS when compared with non-ketamine users (NKUS and HC, see Table 2 and Figure 1A). There were no areas that showed reduced activation for chronic ketamine users. Also, ketamine cue-induced craving showed much more intensive and widespread cortical activation in two regions: anterior cingulate cortex and the precuneus (alphaSim corrected *p* < 0.005) when compared with smoking and sexual cues (see Table 2 and Figure 1). In ROI based association analysis, we found no significant association between ketamine craving scales and ketamine cue-induced brain activation.

#### Functional Activation Analyses: Smoking Craving

In response to smoking-related films, we only observed greater brain regions of activation in the right frontal cortex (precentral gyrus) in chronic smokers (KUS and NKUS) when compared with non-smokers (HC) (see Table 2 and Figure 1B). In ROI-based association analysis, we found no significant association between smoking craving scales and smoking cue-induced brain activation.

#### Functional Activation Analyses: Sexual Cues

For sexual films, increased brain regions of activation in the left cerebellum and middle temporal cortex were observed in non-ketamine users (NKUS and HC) when compared with chronic ketamine users (KUS) (see Table 2 and Figure 1C). In other words, KUS showed reduced activation in those two regions.

#### Functional Activation Analyses: Ketamine Minus Smoking Cues

In order to explore the unique and relatively strong brain activation of ketamine cues, the following analyses has been applied: ketamine cue versus another substance (i.e., smoking/nicotine) cue for KUS compared to the same contrast for NKUS. In respect to ketamine minus smoking cues, we only observed greater brain regions of activation in the left inferior parietal cortex, posterior congulate/precuneus, and left middle temporal cortex in KUS when compared with NKUS (see Table 2 and Figure 1D).

## Discussion

To the best of our knowledge, this is the first study using fMRI to examine the brain regional activation associated with ketamine use, cigarette smoking, and sexual visual cues in a sample of KUS (with ketamine and nicotine dependence, KUS), NKUS (only with nicotine dependence, NKUS) and non-ketamine use non-smoking HC. The present study found that KUS showed significant increased activation mainly in the anterior cingulate cortex and precuneus in response to ketamine cues. Smokers (KUS and NKUS) showed higher activation in the right precentral frontal cortex in response to smoking cues. None-ketamine users (NKUS and HC) showed significant increased activation of cerebellum and middle temporal cortex while viewing sexual cues.

As for *ketamine cues*, this study revealed a wide distribution of brain regions that showed significant greater activation in the ketamine users (KUS) as the ketamine cues were being viewed. Besides those two mainly activated regions (i.e., the anterior cingulate cortex and precuneus), the cingulate gyrus, left inferior parietal cortex, right posterior cingulate, left occipital cortex (lingual gyrus), and right parietal cortex (supramarginal gyrus) were also activated during ketamine craving in KUS when compared with non-ketamine users (NKUS and HC). When compared with smoking, ketamine cue-induced craving was more intensive and widespread, which is consistent with our hypothesis that ketamine use cue-elicited craving of brain activation will be stronger and wider than that of smoking cue-elicited craving. For ketamine minus smoking cues, we observed only greater brain regions of activation (in the left inferior parietal cortex, posterior congulate/precuneus, and left middle temporal cortex) in KUS when compared with NKUS. Higher levels of sensation seeking and novelty-seeking in several classes of addiction (such as heroin and ketamine) in comparison with others (such as alcohol and tobacco) (27) may partly explain why stronger activation occurred during ketamine cues. It is plausible to conclude, however, that the widespread and strong regional (mostly in the limbic system) activations induced by ketamine use in the present study reflect the unique circuitry of ketamine users. A further understanding of these uniquely large responses (i.e., PPI or DCM connectivity analysis) may cast new light on the concept of ketamine dependency and how chronic ketamine use affects normal brain systems for desire, leading to its dependence.

Activation of the anterior cingulate cortex has been reported in cigarette smoking (12, 28) and other substances, such as cocaine (23, 29) and alcohol (30) induced craving. Additionally, this activation is seen in non-substance abuse such as internet gaming (31) induced craving, and has been thought to play a role in drug-seeking behavior (32), cognitive and emotional process (33). The current study further supports an important role for the anterior cingulate cortex in craving—one of the key factors resulting in relapse.

The precuneus, a medial prefrontal-mid-parietal neural network with the association of cortical and subcortical structures, is linked with visual-processing, attention, as well as integrates the related memory and complexity of behavioral specializations (34). While viewing ketamine cues, our study showed activation of the precuneus in KUS compared with NKUS and HC. This finding is also consistent with the previously documented cue-induced craving research in alcohol dependent subjects (35) and internet addictions (31, 36), which suggests a role of the precuneus in cue-induced craving.

In response to *cigarette smoking cues*, this study observed greater activation only in the right precentral frontal cortex in smokers (both KUS and NKUS) when compared with non-smokers (HC). Smoking induced brain activation was found in reward-related brain areas (frontal cortex) in the processing of other smoking-related stimuli (8, 37, 38). However, previous similar studies reported extended regional activation (7–11, 39). The severity of nicotine dependence (i.e., heavily dependent smokers’ craving was more stable than moderately dependent smokers) (40), and even menstrual cycle phase (41) may have resulted in mixed findings. Unfortunately, in the present study, we did not assess both factors. Besides a different design, participants and stimuli are possible factors for mixed findings. Furthermore, our study did not show any differences of smoking cue-induced brain activation between chronic KUS and NKUS.

For *sexual cues*, NKUS and HC showed significant increased activation of cerebellum and middle temporal cortex while viewing sexual cues when compared with KUS, which means chronic ketamine users showed hypo-activation in those two regions. These results may partly reflect the neural basis for the reduced ability of ketamine-dependent subjects to experience sexual pleasure due to their sexual dysfunction. Cocaine users also revealed decreased brain activation induced by watching sexual films (23), and treatment-seeking male cocaine patients showed “unseen” sexual cues elicited strong limbic activations (42). The hypo-activation by natural stimuli (sexual cues) in drug users may suggest its role in drug dependence, treatment, and relapse.

### Limitation

An earlier fMRI study with cocaine users (23) reported that brain response to cocaine cues was stronger than that to sexual cues. From this point of view, we should attempt to directly compare the magnitude of the brain activation associated with cues for a natural (sexual) reward and drug (ketamine or nicotine) reward. However, history of ketamine use, smoking, and sexual experience are uncontrolled variables in the present study. It is difficult to equate the cues on a critical dimension, i.e., their relevance for the individuals’ unique learning history, even when the cue types are otherwise approximately equated on visual characteristics (brightness, hue, shape, orientation, complexity, etc.). Thus, we did not directly compare the brain response to them. Further preclinical fMRI research could offer a novel alternative for establishing and directly comparing brain activation to cue categories for natural and drug rewards. In addition, although the study showed no differences of brain activation during smoking cues between KUS and NKUS, we need to admit that only ketamine users’ abstinence from ketamine use and smoking has been controlled, while smokers’ abstinence from smoking was based only on their self-reported answer.

## Conclusion

In conclusion, KUS showed intense and widespread cortical activation in anterior cingulate cortex and precuneus when they were viewing ketamine cues. Smokers (KUS and NKUS) showed higher activation in right precentral frontal cortex when compared with non-smokers. In contrast with smoking cues, ketamine cues induced much stronger and widespread brain activation. Based on the common symptom of sexual dysfunction, ketamine users (KUS) showed lower activation in cerebellum and middle temporal cortex compared with non-ketamine users (NKUS and HC) in response to sexual cues, which may partly reflect the neural basis of sexual dysfunction. These findings show clearly the engagement of distinct neural circuitry for drug-related stimuli in chronic ketamine users. While smokers (both KUS and NKUS) showed overlapping differences in activation for smoking cues, the former group showed a specific neural response to relevant (i.e., ketamine-related) cues. In particular, the heightened response in anterior cingulate cortex may have important implications for how attentionally salient such cues are in this group.

### Clinical Implications

Cue-induced drug craving is often cited as a major determinant in drug relapse. Identifying patterns of whole-brain activation associated with cue categories for natural and drug rewards may have clinical implications; for example, helping optimize therapeutic interventions that block craving and attenuate compulsive drug-seeking behavior. Encouragingly, this implicates that cue exposure treatment [Cue Exposure Therapy (43)], with multiple methodological innovations for addiction may be a speculative used therapy in clinical settings.

## Ethics Statement

This study was approved by The Second Xiangya Hospital of Central South University Review Board (No. S054, 2008). All human subjects were fully informed about the measurement and MRI scanning procedures in the study. Written informed consent was given and obtained by all subjects. The study included chronic ketamine users, smokers, and none-smokers. It did not include any specifically vulnerable populations.

## Author Contributions

All of the authors contributed to the study conception and design, interpretation of findings, and manuscript preparation and revision. YL originated the study and drafted the manuscript. JT conducted the statistical analyses and revised the manuscript. WH advised on the statistical analysis, interpretation of findings, and reviewed drafts of the manuscript.

## Conflict of Interest Statement

The authors declare that the research was conducted in the absence of any commercial or financial relationships that could be construed as a potential conflict of interest.
