# Supplementary material for: Cue-Induced Brain Activation in Chronic Ketamine-Dependent Subjects, Cigarette Smokers, and Healthy Controls: A Task Functional Magnetic Resonance Imaging Study
Source: Front Psychiatry. 2018 Mar 21;9:88. doi: 10.3389/fpsyt.2018.00088 (PMC5872489; doi:10.3389/fpsyt.2018.00088)
Supplement: Supplementary file 1 [file Presentation_1.PDF]

**Figure S1: fMRI task design**

**All 9 films (total 22 minutes and 30 seconds)**

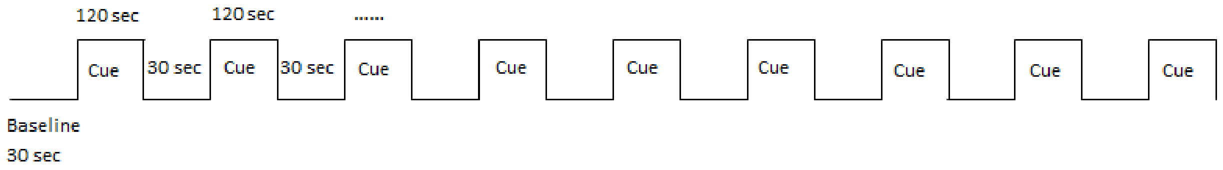


There were nine 120-sec-long films, including three 2-minute sexual related visual films selected from Asian movies of heterosexual activity, three 2-minute ketamine related films made by ketamine user and three 2-minute smoking related films made by otherwise healthy smokers. Each film was randomly presented for 2 minutes, followed by a black screen for 30 seconds.

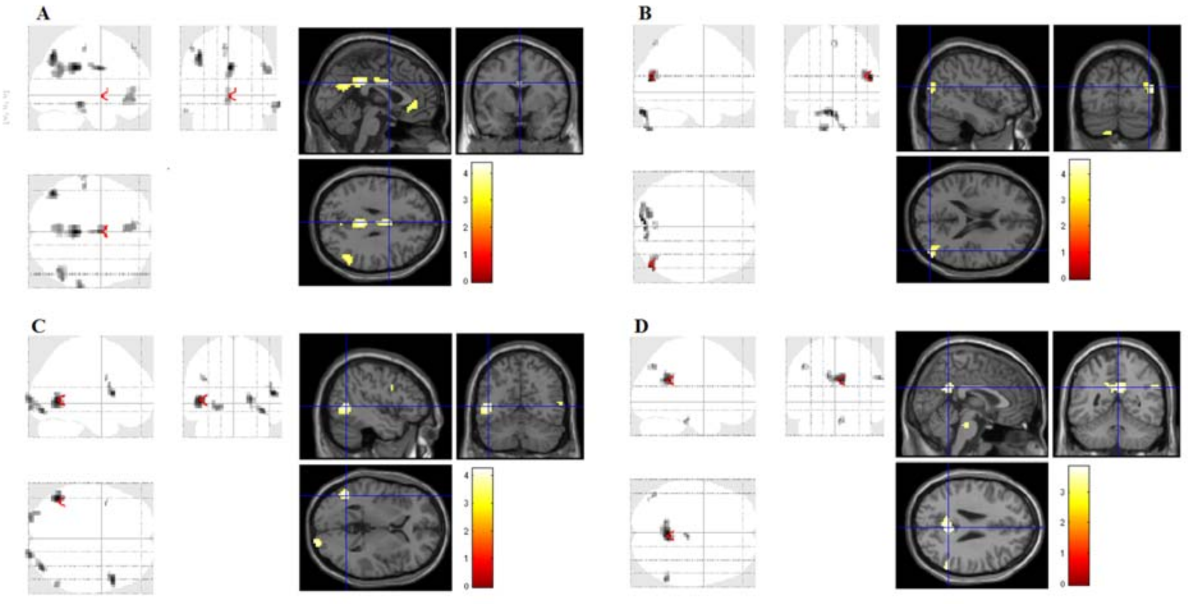


**Figure S2. Increased brain regions of activation by different cues (male subjects only)**

Increased brain regions of activation in chronic ketamine users during exposure to ketamine use related films when compared with control subjects (smokers and nonsmokers, figure S2. A); Increased brain regions of activation in chronic smokers (chronic ketamine users were also chronic smokers) during

exposure to smoking related films when compared with nonsmokers (figure S2. B); increased brain regions of activation in control subjects (smokers and nonsmokers) during exposure to sexual films when compared with chronic ketamine users (figure S2. C); in respects to ketamine cue minus smoking cue, increased brain regions of activation in chronic ketamine users when compared with chronic smokers (figure S2. D).

AlphaSim corrected $p < 0.005$.
